# Evaluation of fluid responsiveness with dynamic superior vena cava collapsibility index in mechanically ventilated patients

**DOI:** 10.1186/s13741-023-00298-z

**Published:** 2023-04-10

**Authors:** Yaru Li, Luyang Jiang, Lu Wang, Dou Dou, Yi Feng

**Affiliations:** Department of Anesthesiology, Peking University People’s Hospital, 11 Xi Zhi Men South Street, Beijing, 100044 China

**Keywords:** Fluid responsiveness FR, Transesophageal echocardiography TEE, Superior vena cava SVC, Stroke volume variation SVV

## Abstract

**Background:**

This study aimed to evaluate the predictive accuracy of the superior vena cava collapsibility index measured by transesophageal echocardiography and compare the index with stroke volume variation measured by FloTrac™/Vigileo™ in mechanically ventilated patients.

**Methods:**

In the prospective study, a total of 60 patients were enrolled for elective general surgery under mechanical ventilation, where all patients received 10 ml/kg of Ringer’s lactate. Five kinds of related data were recorded before and after the fluid challenge, including the superior vena cava collapsibility index (SVC-CI), the ratio of E/e’, cardiac index (CI), stroke volume variation (SVV), and central venous pressure (CVP). Based on the collected data after the fluid challenge, we classified the patients as responders (FR group) if their CI increased by at least 15% and the rest were non-responders (NR).

**Results:**

Twenty-five of 52 (48%) of the patients were responders, and 27 were non-responders (52%). The SVC-CI was higher in the responders (41.90 ± 11.48 vs 28.92 ± 9.05%, *P* < 0.01). SVC-CI was significantly correlated with △CI _FloTrac_ (*r* = 0.568,* P* < 0.01). The area under the ROC curve (AUROC) of SVC-CI was 0.838 (95% CI 0.728 ~ 0.947, *P* < 0.01) with the optimal cutoff value of 39.4% (sensitivity 64%, specificity 92.6%). And there was no significant difference in *E*/*e*’ between the two groups (*P* > 0.05). The best cutoff value for SVV was 12.5% (sensitivity 40%, specificity 89%) with the AUROC of 0.68 (95% CI 0.53 ~ 0.826, *P* < 0.05).

**Conclusions:**

The SVC-CI and SVV can predict fluid responsiveness effectively in mechanically ventilated patients. And SVC-CI is superior in predicting fluid responsiveness compared with SVV. The *E*/*e*’ ratio and CVP cannot predict FR effectively.

**Trial registration:**

Chinese clinical trial registry (ChiCTR2000034940).

## Introduction

Appropriate administration of fluid is the main treatment in the perioperative period. According to the Frank-Staring curve, increased preload can increase the patient’s stroke volume until it reaches the flat phase of the curve (Cecconi et al. 2015; Marik 2009; Frank O; Starling 1918). Fluid overloading might be deleterious due to cause systemic and pulmonary edema. As a result, assessing FR accurately is critical to avoid fluid overloading. FR was defined by a 15% increase of the CO, CI, or SV after fluid administration (Cecconi 2014), while 50% of patients are fluid responders in ICU and operation rooms. Considering this, there are some dynamic indices proposed for predicting FR such as stroke volume variation and pulse pressure variation (Michard et al. 2002; Cherpanath et al. 2016).

Recently, the development of Point-of-Care Ultrasound (POCUS) makes it possible to predict FR visually. Moreover, perioperative transesophageal echocardiography (TEE) or transthoracic echocardiography (TTE) can provide real-time hemodynamic monitoring. Several studies found that respiratory diameter variation of great veins connected to the right atrial chamber might predict FR effectively (Bubenek-Turconi et al., 2020; Cheng et al. 2019). Under mechanical ventilation, the superior vena cava (SVC) expands or collapses regularly. During inhalation, the intrathoracic pressure will increase, which causes the SVC directly compressed and collapsed as an intrathoracic vein. On the contrary, SVC expands during expiration (Vieillard-Baron et al. 2004). And the periodic changes in the SVC diameter are even more evident in hypovolemic patients.

In addition, the ratio of *E*/*e*’ that estimate the pulmonary capillary wedge pressure (PCWP) provides another way to quantitatively evaluate the LV preload, where the *E* velocity refers to the peak early filling velocity of rapid trans-mitral flow as the mitral valve opens during early diastole. And the *e*’ is the mitral annular tissue early diastole velocity (Diwan et al. 2005.)

The primary objective of this study was to assess the predictive accuracy of SVC-CI and the ratio of E/e’ measured by TEE to predict FR in mechanically ventilated patients, and the secondary objective was to compare the predictive capacity of those TEE variables with conventional indices including SVV and CVP.

## Methods

### Study design

The prospective diagnostic study was conducted in the department of anesthesiology of People’s Hospital of Peking University and was approved by the Institutional Review Board of our institution (Ethics Committee of Peking university people's hospital 2020PHB139-01). And the study was registered in the Chinese clinical trial registry (ChiCTR2000034940). Written informed consent was obtained from all participants. The patients who underwent general anesthesia with tracheal intubation for abdominal surgery were consecutively included. Inclusion criteria included the age of 18–70 years old, the ASA of I-III, and the NYHA of I-II grade, while the exclusion criteria included TEE contradictions such as gastroduodenal ulcer, the history of esophagus operation, esophagus fundus ventricular varication, arrhythmia, and susceptive heart dysfunction including left ventricle EF < 55%, average *E*/*e*’ > 14 or *e*’ _average_ < 9 cm/s at baseline, and valvular diseases.

Upon arrival in the operating room, all patients were monitored with pulse oximetry and electrocardiograph, and radial artery catheterization which was connected to the FloTrac™/Vigileo™ (Edwards Lifesciences, USA). And anesthesia induction was done with midazolam (1 mg), sufentanil (0.25 μg/kg), etomidate (0.3 mg/kg), and rocuronium (0.6–1 mg/kg) and maintained with sevoflurane inhalation. All patients were continuously monitored with BIS (range of 40–60) and mechanically ventilated in volume-controlled ventilation under *V*_*T*_ of 8 ml/kg, respiratory rate of 12 breath/min, where no PEEP was applied.

After induction, the TEE probe (6TC-RS GE Medical Horton, Norway) was inserted orally. During the whole measurement, all patients were maintained in the supine position; meanwhile, neither procedures including pneumoperitoneum were performed nor vasoactive drugs were used.

### Data collection

During the experiments, we collect the necessary data including the following:

- Basic hemodynamic data: *MAP*,* HR*, and *CVP*;
- Functional hemodynamic data using FloTrac: *CI* and *SVV*;
- Echocardiographic data using TEE: *SVC-CI*,* E/e’*;
- SVC-CI (via *M*-mode), *E* velocity (via pulse wave doppler), *e*’ (*e*’ = *e*’_lateral_ + *e*’ _septal_/2, via tissue Doppler from both lateral and septal side of mitral annular);

### Fluid responsiveness

Fluid challenge: A fluid challenge was conducted with 10 ml/kg of a Ringer’s lactate for 30 min.

△CI_FloTrac_ was calculated as follows: △CI_FloTrac_ = (CI_after_ -CI_baseline_)/CI_baseline_ × 100%. Patients were classified as responders (FR group: △CI _FloTrac_ ≥ 15%) and non-responders (NR group:△CI_FloTrac_ < 15%).

### Data measurements

#### SVC-CI measurement

**Fig. 1:**
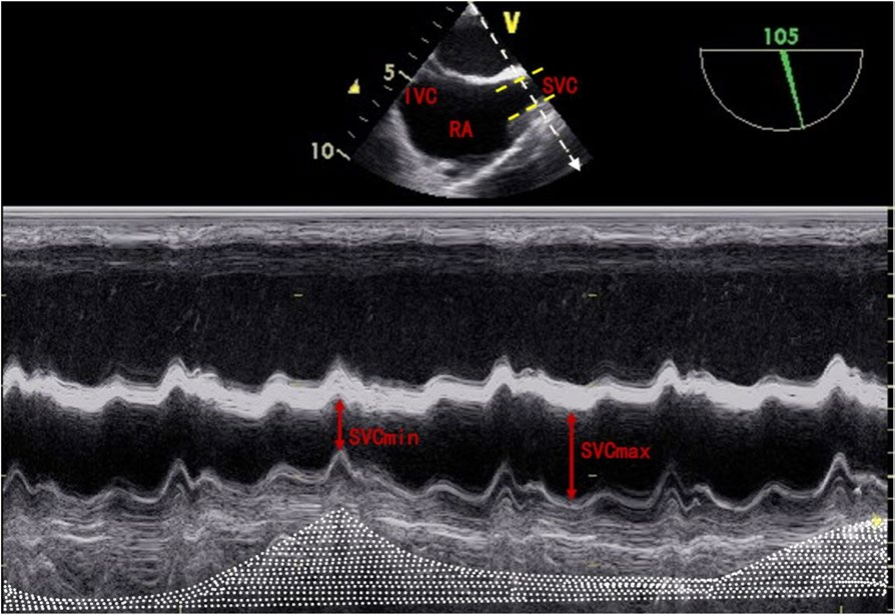
Measurement of the SVC diameter, take the M-mode cursor (white arrow) perpendicular to the SVC (yellow dotted line) in mid-bicaval view. The mechanical ventilation cycle was indicated by spontaneous airway pressure wave (white dotted area). SVC, superior vena cava; RA, right atrial; IVC, inferior vena cava; SVCmin, minimum diameter of SVC; SVCmax, maximum diameter of SVC

After tracheal intubation, we inserted the TEE probe into the mid-esophagus (ME) position. During that the transducer angle of the probe was rotated forward from 90 to 110° to obtain the ME bicaval view, where the superior vena cava (SVC) and the right atrium (RA) can be observed well. The SVC diameter was measured at the position of approximately 2 cm from the junction with RA using the M-mode, where we move the M-mode cursor to the junction and measure the perpendicular distance of the SVC to obtain the inner diameter within a single respiratory cycle (Fig. 1) (Hahn et al. 2013). The maximum and minimum diameter over a single respiratory cycle were collected. After that, SVC-CI was calculated as follows:

SVC-CI = (SVC_max_ − SVC_min_)/SVC_max_ × 100%. Echocardiographic variables were derived from the US machine (Vivid 7 Pro, GE Vingmed Ultrasound AS, Horten, Norway). All measurements were made three times and the average was used for statistical analysis.

#### E/e’ measurement

At the ME four-chamber view, position the pulse wave (PW) Doppler sample volume between mitral leaflet tips and adjust the sample volume to align with the blood flow, then obtain the optimal image of the *E* wave. At the same view, position the tissue Doppler (TDI) sample volume both at lateral and septal basal regions of mitral annular to acquire *e*’_lateral_ and *e*’_septal_. The average *e*’ velocity can be computed: *e*’_average_ = (*e*’_septal_ + *e*’_lateral_)/2 (Lang et al. 2015).

All measurements were performed by a national board of qualified echocardiography anesthesiologist strictly following the relevant guidelines [Hahn et al. 2013; Nagueh et al. 2016]. Fluid administration and the statistics were performed by two other individuals, and the three researchers were independent of each other.

### Statistical analysis

For continuous variables, data were expressed as mean ± SD (normality distribution) or median with interquartile range (non-normality distribution). For categorical variables, percentages were calculated and the normality distribution was assessed by the Shapiro–Wilk normality tests, and comparisons of percentages were performed with Fisher’s exact test. The differences between the FR group and NR group were assessed using the Mann–Whitney *U* test or Student’s *t* test.

To determine the ability to predict FR, receiver operating characteristics (ROC) curves were generated and the area under the ROC curve (AUROC) was calculated. All *P* values were two-tailed, and a *P* value < 0.05 was considered significant. All statistical analyses were performed with IBM SPSS Statistics 26.0 (IBM, Somers, NY, USA).

### Sample size

Medcalc software (Windows 19.4, Ostend, Belgium) was used to calculate the sample size. According to the pilot study, we assume the AUROC of SVC-CI was 0.75, with an *α* error of 0.05 and power of 0.9, and the sample size in the FR/NR group was the same. Twenty-six patients were required for each group. Considering dropouts, we planned to recruit 60 patients finally.

## Results

### Patients’ characteristics

**Fig. 2:**
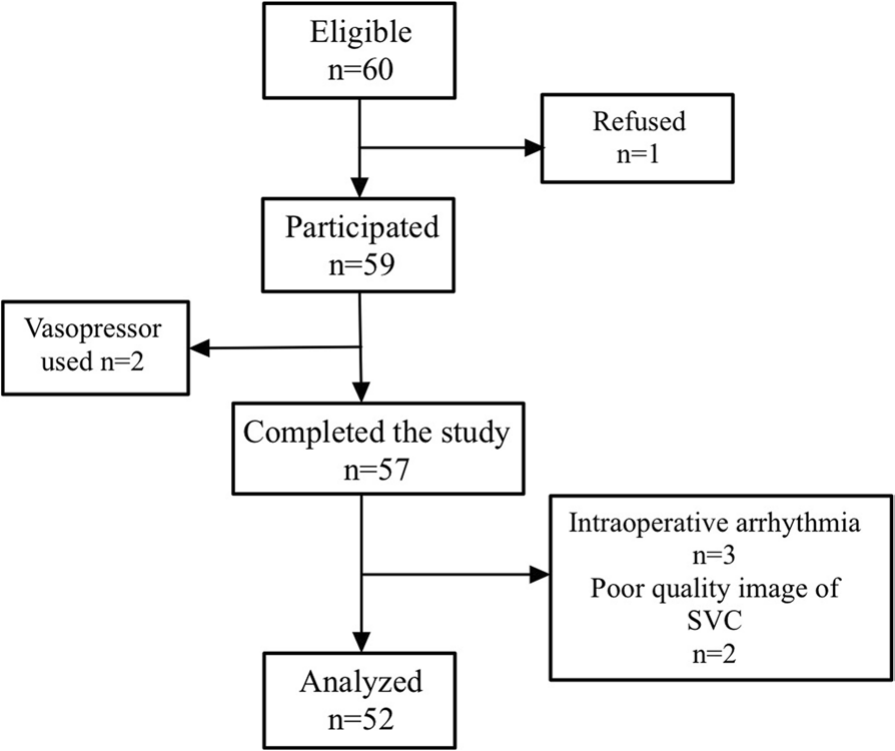
Flowchart of enrollment and outcomes SVC superior vena cava

Sixty patients were enrolled over 8 months (from August 2020 to May 2021) in the study, where 8 patients were excluded due to the following reasons: consent refused (1 case), vasopressors used due to hypotension (2 cases), arrhythmia (3 cases), and poor SVC image (1 case). The flowchart of the enrollment is illustrated in Fig. 2. Consequently, 52 patients completed the study including colorectal surgery (*n* = 22), hepatectomy (*n* = 12), and pancreaticoduodenectomy (*n* = 18), where there were a total of 25 fluid responders and 27 non-responders. The general characteristics of all patients and comparisons between the FR and NR groups are shown in Table 1. While no difference was found between the two groups. All results between these two groups before and after the fluid challenge are reported in Table 2.

**Table 1:** Baseline characteristics between the FR and NR groups

Group	Overall (*n* = 52)	FR (*n* = 25)	NR (*n* = 27)
Gender (M/F)	24/28	12/13	12/15
Age [* M*(Q) *y*]	57 (49, 63)	58 (39, 63)	56 (51, 65)
BMI (*x* ± *s* kg/m^2^)	23.7 ± 3.6	24.6 ± 4.2	22.9 ± 3.0
ASA (I/II/III)	20/28/4	10/13/2	10/15/2
NYHA (I/II)	23/29	11/14	12/15

*Abbreviations*: *BMI*, body mass index; *ASA*, American Society of Anesthesiologists; *NYHA*, New York Heart Association

**Table 2:** Hemodynamic and echocardiographic data before and after fluid challenge

	FR (*n* = 25)		NR (*n* = 27)	
	Baseline	After	Baseline	After
MAP (mmHg)	80.3 ± 12.0	82.2 ± 12.7	86.2 ± 14.6	79.0 ± 13.3
HR (Bpm)	68.4 ± 12.9	65.7 ± 12.2	72.7 ± 15.3	59.2 ± 9.0
SVC-CI (%)	41.9 ± 11.5^a^	29.5 ± 8.4^b^	28.9 ± 9.0	24.9 ± 9.7
SVV (%)	11.6 ± 3.2^a^	6.7 ± 3.2^b^	9.5 ± 2.9	7.0 ± 2.5
E/e’	7.27 ± 2.32	9.01 ± 2.97	8.76 ± 3.28	8.90 ± 4.65
CVP (mmHg)	5.7 ± 3.2	7.8 ± 4.3^b^	5.8 ± 3.1	8.1 ± 4.5^c^

*Abbreviations*: *MAP*, mean arterial pressure; *HR*, heart rate; *SVC-CI*, superior vena cava collapsibility index; *SVV*, stroke volume variation; *CVP*, central venous pressure

^a^*P* < 0.05 compared with non-responders, ^b^*P* < 0.05 compared with baseline in the FR group, ^c^*P* < 0.05 compared with baseline in the NR group

### Echocardiographic data

#### SVC-CI analysis

**Fig. 3:**
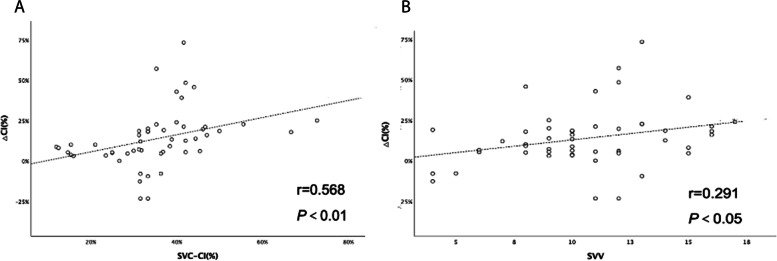
**A** Relationship between △CI_FloTrac_ and basic SVC-CI in all patients. **B** Relationship between △CI_FloTrac_ and basic SVV in all patients

Basic SVC-CI was correlated with △CI_FloTrac_ (*r* = 0.568, *P* < 0.01; Fig. 3A). Specifically, the basic SVC-CI was higher in the FR compared to the NR group (41.90 ± 11.48 s vs 28.92 ± 9.05 s *P* < 0.01 = . And SVC-CI reduced more significantly in the FR group compared with the NR group after the fluid challenge.

#### E/e’ analysis

No correlation was found between E/e’ and △CI _FloTrac_ (*P* > 0.05), and there was no significant difference in E/e’ between the two groups (*P* > 0.05).

### Conventional hemodynamic data

SVV was correlated slightly with △CI _FloTrac_ (*r* = 0.291,* P* < 0.05A; Fig. 3B = and the SVV in the FR group was higher than the NR group either (11.3 ± 3.18 vs 9.52 ± 2.94 *P* < 0.05). CVP was not correlated with △CI_FloTrac_. There was no difference in the basic CVP, HR, and MAP between the two groups. The HR decreased and CVP increased (*P* < 0.05) after the fluid challenge.

### ROC curve analysis

**Fig. 4:**
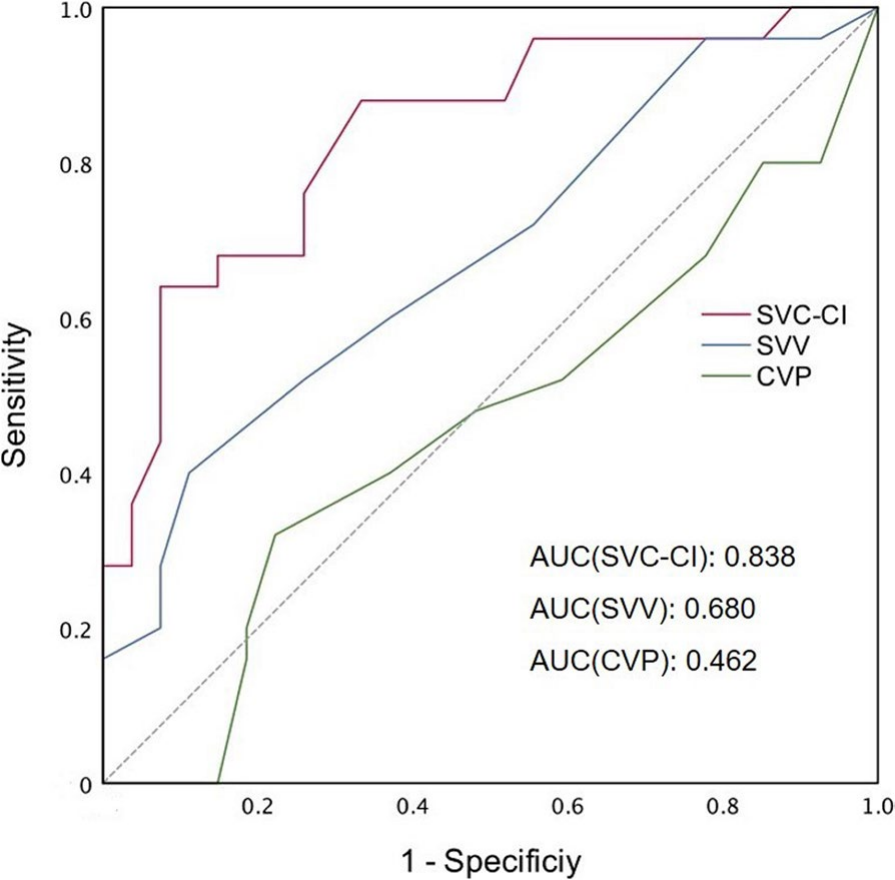
Area under the receiver operating characteristic (ROC) curves to predict fluid responsiveness at baseline. SVC-CI, superior vena cava collapsibility index; SVV, stroke volume variation; CVP, central vena pressure; ROC, receiver operating characteristics

The best cutoff value of SVC-CI was 39.4% with 64% sensitivity and 92.6% specificity. The AUROC of SVC-CI was 0.838 (95% CI 0.728 ~ 0.947, *P* < 0.01). SVV had a sensitivity of 40% and a specificity of 89% to predict FR at a cutoff value of 12.5%, and the AUROC was 0.68 (95% CI 0.53 ~ 0.826, *P* < 0.05). The AUROC of CVP was 0.462 (*P* > 0.05). The results of the ROC analysis are shown in Fig. 4.

## Discussion

Our prospective study found that both SVC-CI and SVV were reliable to predict FR in mechanically ventilated patients and SVC-CI showed better accuracy than SVV regarding the area under the curve of ROC. However, the value of *E*/*e*’ and CVP to assess the FR was doubtful.

FR has been variably defined by an increase of 10–15% in SV, CO, or CI, where CI is the most important index to eliminate the confounding factors including HR and the weight (Messina et al. 2018a, b). Another controversy in the definition of FR is the volume of the fluid administration. About 77% of our subjects underwent bowel preparation for abdominal surgery, and all patients were applied preoperative fasting for 10 h more. Considering the type of surgery and the relatively long fasting period, we classified patients with an increasing in CI at least of 15% after 10 ml/kg of Ringer’s lactate as fluid responders (Kang et al. 2016). As a result, the ratio of FR to NR was approximately 1:1, which was consistent with previous studies (Lee et al. 2012; Lee et al. 2011; Kang et al. 2016).

The SVC diameter is determined by the blood volume and intrathoracic pressure, which depends on positive-pressure ventilation in mechanically ventilated patients. When the volume is insufficient, suddenly increased intrathoracic pressure during inhalation exceeding the inner-vascular pressure will cause the SVC collapses consequently. Accordingly, the collapse of the SVC might reflect the blood volume in ventilated patients. Initially, Vieillard-Baron A’s classic study (Vieillard-Baron et al. 2004) defined FR as an 11% increase of CI and the optimal cutoff value of SVC-CI for predicting FR was 36%. In our study, we found the basic SVC-CI of the FR group was greater than the NR group significantly. Moreover, the reduction in SVC-CI after fluid challenge in the FR group was greater than that in the NR group, which indirectly revealed that the fluid administration cannot increase the effective circulating blood volume in the NR group. None of the subjects had any cardiopulmonary disease or were treated with vasopressors in our study, so it is convincing that lung compliance and cardiac contraction had less effect on the experiment results.

SVV is considered to be reliable for predicting FR. The cutoff value for SVV to predict FR was 12.5% in our research, which is close to the 13% threshold recommended by FloTrac instruction. However, it has some acknowledged limitations. For instance, SVV is not suitable for some cases such as pneumoperitoneum, arrhythmia, spontaneous breathing, and vasopressor used (Messina et al. 2018a, b; Alvarado Sanchez et al. 2018). On the other hand, the accuracy of SVV also depends on the waveform of the peripheral radial artery. Compared with SVV, SVC is not affected by the above factors.

The ratio of *E*/*e*’ is considered to be reliable to estimate PCWP, which was proved being able to reflect preload (Meersch et al. 2016). However, our study showed that *E*/*e*’ cannot discriminate FR effectively, which might be because PCWP represents not only LV diastolic function but also systolic function, which however was not affected by even 10 ml/kg fluid challenge in subjects with normal heart function (Porter et al. 2015).

Consistent with previous literature, there is no evidence suggesting that CVP could discriminate FR effectively.

Due to the study design, the TEE probe was inevitably kept placed in the patients’ bodies for a duration for providing some necessary real-time information. However, the long-time goal is incorporating the SVC-CI measurement into bedside POCUS monitoring to evaluate the FR of critical patients non-invasively and quickly. We assume that the greater the SVC-CI, the greater the increase of CO after rapid infusion. If the patient’s basic SVC-CI is less than 39.4%, the rapid infusion will not increase the cardiac output with potentially harmful effects.

There are several limitations to our study. First, to avoid the potential deleterious effect of the rapid fluid administration, we excluded the patients order than 70 years old who were actually more necessary to assess FR. Second, due to the study design, the influence of pneumoperitoneum or body position on assessing FR was not discussed. Third, although TEE is a minimally invasive procedure while some TEE-related complications range from 0.2 to 0.5% were still reported (Daniel et al. 1991).

## Conclusion

The superior vena cava collapsibility index (SVC-CI) and SVV can predict FR effectively in mechanically ventilated patients. And SVC-CI is superior in predicting FR compared to SVV in our study. The *E*/*e*’ ratio and CVP cannot predict FR effectively.

## Data Availability

The datasets used and/or analyzed during the current study are available from the corresponding author on reasonable request.

## Abbreviations

CVP: Central venous pressure
CI: Cardiac index
SVV: Stroke volume variation
FR: Fluid responsiveness
AUROC: Area under the receiver operating characteristic curve
TEE: Transesophageal echocardiography

## References

[CR1] Alvarado Sanchez JI, Amaya Zuniga WF, Monge Garcia MI (2018). Predictors to intravenous fluid responsiveness. J Intensive Care Med.

[CR2] Bubenek-Turconi ŞI, Hendy A, Băilă S, Drăgan A, Chioncel O, Văleanu L, Moroșanu B, Iliescu VA (2020). J Clin Monit Comput.

[CR3] Cecconi M, De Backer D, Antonelli M, Beale R, Bakker J, Hofer C, Jaeschke R, Mebazaa A, Pinsky MR, Teboul JL, Vincent JL, Rhodes A (2014). Consensus on circulatory shock and hemodynamic monitoring. Task force of the European Society of Intensive Care Medicine. Intensive Care Med.

[CR4] Cecconi M, Hofer C, Teboul JL, Pettila V, Wilkman E, Molnar Z, Della Rocca G, Aldecoa C, Artigas A, Jog S, Sander M, Spies C, Lefrant JY, De Backer D, Investigators F, Group ET (2015). Erratum to: fluid challenges in intensive care: the FENICE study: a global inception cohort study. Intensive Care Med.

[CR5] Cheng Z, Yang QQ, Zhu P, Feng JY, Zhang XB, Zhao ZB (2019). Transesophageal echocardiographic measurements of the superior vena cava for predicting fluid responsiveness in patients undergoing invasive positive pressure ventilation. J Ultrasound Med.

[CR6] Cherpanath TG, Hirsch A, Geerts BF, Lagrand WK, Leeflang MM, Schultz MJ, Groeneveld AB (2016). Predicting fluid responsiveness by passive leg raising: a systematic review and meta-analysis of 23 clinical trials. Crit Care Med.

[CR7] Daniel WG, Erbel R, Kasper W, Visser CA, Engberding R, Sutherland GR, Grube E, Hanrath P, Maisch B, Dennig K (1991). Safety of transesophageal echocardiography. A multicenter survey of 10,419 examinations. Circulation..

[CR8] Diwan A, McCulloch M, Lawrie GM, Reardon MJ, Nagueh SF (2005). Doppler estimation of left ventricular filling pressures in patients with mitral valve disease. Circulation.

[CR9] Frank O (1895). Zur Dynamik Des Herzmuskels Z Biol.

[CR10] Hahn RT, Abraham T, Adams MS, Bruce CJ, Glas KE, Lang RM, Reeves ST, Shanewise JS, Siu SC, Stewart W, Picard MH (2013). Guidelines for performing a comprehensive transesophageal echocardiographic examination: recommendations from the American Society of Echocardiography and the Society of Cardiovascular Anesthesiologists. J Am Soc Echocardiogr.

[CR11] Kang WS, Oh CS, Park C, Shin BM, Yoon TG, Rhee KY (2016). Diagnosis accuracy of mean arterial pressure variation during a lung recruitment maneuver to predict fluid responsiveness in thoracic surgery with one-lung ventilation. Biomed Res Int.

[CR12] Lang RM, Badano LP, Mor-Avi V, Afilalo J, Armstrong A, Ernande L, Flachskampf FA, Foster E, Goldstein SA, Kuznetsova T, Lancellotti P, Muraru D, Picard MH, Rietzschel ER, Rudski L, Spencer KT, Tsang W, Voigt JU. "Recommendations for cardiac chamber quantification by echocardiography in adults: an update from the American Society of Echocardiography and the European Association of Cardiovascular Imaging". J Am Soc Echocardiogr. 2015;28(1):1-39 e14. 10.1016/j.echo.2014.10.003 25559473

[CR13] Lee JH, Jeon Y, Bahk JH (2011). Pulse-pressure variation predicts fluid responsiveness during heart displacement for off-pump coronary artery bypass surgery. J Cardiothorac Vasc Anesth.

[CR14] Lee JY, Park HY, Jung WS, Jo YY, Kwak HJ (2012). Comparative study of pressure- and volume-controlled ventilation on stroke volume variation as a predictor of fluid responsiveness in patients undergoing major abdominal surgery. J Crit Care.

[CR15] Marik PE (2009). Techniques for assessment of intravascular volume in critically ill patients. J Intensive Care Med.

[CR16] Meersch M, Schmidt C, Zarbock A (2016). Echophysiology: the transesophageal echo probe as a noninvasive Swan-Ganz catheter. Curr Opin Anaesthesiol.

[CR17] Messina A, Pelaia C, Bruni A (2018). Fluid challenge during anesthesia: a systematic review and meta-analysis. Anesth Analg.

[CR18] Messina A, Pelaia C, Bruni A, Garofalo E, Bonicolini E, Longhini F, Dellara E, Saderi L, Romagnoli S, Sotgiu G, Cecconi M, Navalesi P (2018). Fluid challenge during anesthesia: a systematic review and meta-analysis. Anesth Analg.

[CR19] Michard F, Teboul JL (2002). Predicting fluid responsiveness in ICU patients: a critical analysis of the evidence. Chest.

[CR20] Nagueh SF, Smiseth OA, Appleton CP, Byrd BF, Dokainish H, Edvardsen T, Flachskampf FA, Gillebert TC, Klein AL, Lancellotti P, Marino P, Oh JK, AlexandruPopescu B, Waggoner AD, Houston T, Oslo N, Phoenix A, Nashville T, Hamilton OC, Uppsala S, Ghent, Liege B, Cleveland O, Novara I, Rochester M, Bucharest R, St. Louis M (2016). Recommendations for the evaluation of left ventricular diastolic function by echocardiography: an update from the American Society of Echocardiography and the European Association of cardiovascular imaging. Eur Heart J Cardiovasc Imaging.

[CR21] Porter TR, Shillcutt SK, Adams MS, Desjardins G, Glas KE, Olson JJ, Troughton RW (2015). Guidelines for the use of echocardiography as a monitor for therapeutic intervention in adults: a report from the american society of echocardiography. J Am Soc Echocardiogr.

[CR22] Starling EH (1918). Linacre lecture on the law of the heart.

[CR23] Vieillard-Baron A, Chergui K, Rabiller A, Peyrouset O, Page B, Beauchet A, Jardin F (2004). Superior vena caval collapsibility as a gauge of volume status in ventilated septic patients. Intensive Care Med.

[CR24] Wu CY, Cheng YJ, Liu YJ, Wu TT, Chien CT, Chan KC (2016). Predicting stroke volume and arterial pressure fluid responsiveness in liver cirrhosis patients using dynamic preload variables: a prospective study of diagnostic accuracy. Eur J Anaesthesiol.

[CR25] Zlicar M, Novak-Jankovic V, Blagus R, Cecconi M (2018). Predictive values of pulse pressure variation and stroke volume variation for fluid responsiveness in patients with pneumoperitoneum. J Clin Monit Comput.

